# Evaluation of 3D Footprint Morphology of Knee-Related Muscle Attachments Based on CT Data Reconstruction: A Feasibility Study

**DOI:** 10.3390/life14060778

**Published:** 2024-06-19

**Authors:** Anne-Marie Neumann, Maeruan Kebbach, Rainer Bader, Guido Hildebrandt, Andreas Wree

**Affiliations:** 1 Institute for Anatomy, Rostock University Medical Center, Gertrudenstraße 9, 18057 Rostock, Germany; anne-marie.neumann@uni-ulm.de; 2 Institute of Molecular and Cellular Anatomy, University of Ulm, Albert-Einstein-Allee 11, 89081 Ulm, Germany; 3 Department of Orthopaedics, Rostock University Medical Center, Doberaner Straße 142, 18055 Rostock, Germany; maeruan.kebbach@med.uni-rostock.de (M.K.); rainer.bader@med.uni-rostock.de (R.B.); 4 Department of Radiotherapy and Radiation Oncology, Rostock University Medical Center, Südring 75, 18059 Rostock, Germany; guido.hildebrandt@med.uni-rostock.de

**Keywords:** muscle attachments, musculoskeletal modeling, knee joint, footprint morphology, CT data

## Abstract

A three-dimensional (3D) understanding of muscle attachment footprints became increasingly relevant for musculoskeletal modeling. The established method to project attachments as points ignores patient-specific individuality. Research focuses on investigating certain muscle groups rather than comprehensively studying all muscles spanning a joint. Therefore, we present a reliable method to study several muscle attachments in order to reconstruct the attachment sites in 3D based on CT imaging for future applications in musculoskeletal modeling. For the present feasibility study, 23 knee-related muscle attachments were CT-scanned postmortem from four nonadipose male specimens. For this, the specific muscle attachments were dissected and marked with a barium sulfate containing paint (60 g BaSO_4_ in 30 mL water and 10 mL acrylic paint). Subsequently, bone geometries and muscle attachments were reconstructed and evaluated from CT datasets. Bone morphology and footprint variations were studied. Exemplarily, variations were high for pes anserinus insertions (mean 56%) and the origins of M. biceps femoris (mean 54%). In contrast, the origins of the vastus muscles as well as the insertion of the Achilles tendon showed low variation (mean 9% and 13%, respectively). Most attachment sites showed variation exceeding the individuality of bone morphology. In summary, the present data were consistent with the few published studies of specific muscle footprints. Our data shed light on the high variability of muscle attachments, which need to be addressed when studying muscle forces and movements through musculoskeletal modeling. This is the first step to achieving a more profound understanding of muscle morphology to be utilized in numerical simulations.

## 1. Introduction

The patellofemoral joint is known to bear some of the highest loads in the human body. It is estimated to carry up to 2.5 to 2.8× the body weight during easy walking compared to, e.g., 1.2× in the foot [1,2]. Furthermore, the patellofemoral load varies considerably during different activities, ranging from 0.6× body weight during walking to more than 8× body weight during a single-leg squat [3]. As such, diffuse knee pain syndromes and knee-related sports injuries are common. Multicausal anterior knee pain (AKP) is associated with muscle or tendon dysfunctions [4]. It is especially found in young athletes. Nearly 40% of adolescent athletes independent of primary sport played, age or sex reported AKP upon provocation [5]. AKP accounts for 20% of female sports injuries compared to 7.5% of male injuries [6]. Despite this, treatment and etiology of this disease are still insufficiently understood. Numerical simulations can help in predicting potential sources for dysfunctions but for reliable subject-specific models and meaningful conclusions accurate musculoskeletal geometry is essential [7,8,9]. Furthermore, musculoskeletal models are frequently used to support preoperative planning to evaluate joint dynamics and to simulate physiological or pathological movements [8,9,10]. Biomechanical engineers and clinicians aim to increase their efforts in building a diverse database of muscle attachment details in relation to various ages, body types and lifestyles of patients. 

Qualitative or descriptive studies of specific muscle attachments and ligaments are invaluable and have progressed understanding of individuality in muscle anatomy [11,12,13]. However, attachment sites on the bone surfaces are often studied exclusively two-dimensionally based on macroscopic images, plain radiographs or magnetic resonance imaging (MRI) [14]. Three-dimensional (3D) characterization of footprints allows for more thorough morphological analysis [15,16]. The study of bone curvature and shape derived from 3D image data allows for reliable estimations of some attachment surfaces on bones, but it lacks single muscle resolution on conjoined attachment sites [17,18]. Furthermore, in statistical shape modeling, the error increases as the attachment size is reduced [19]. Additionally, Carbone et al. [7] introduced a valuable database (TLEM 2.0) which contains data on muscle attachment sites for musculoskeletal modeling based on the dissection of one human specimen. In this regard, Andreassen et al. [20] presented a male and female dataset based on medical images that included muscle geometries and attachment areas. However, these datasets provide no individual differences in the attachment areas.

It is also possible to deploy probabilistic approaches. For this, Fukuda et al. [21] proposed a probabilistic model, where they investigated the differences in muscle attachment areas for the hip muscles based on the dissection of eight human specimens. The individual muscle attachment areas of the hip region were defined using an optical tracker. Based on a probabilistic model with these measurements, specific muscle attachment areas for simulation models can be estimated and mapped using CT-derived models. Although this is a promising approach, there were also outliers in the measurement that had to be removed manually. Herteleer et al. [22] showed the variation of muscle attachment areas in the clavicle. However, they did not analyze the knee-relevant muscle areas or the centroids of the attachment areas. By using a 3D-digitizer and microscribes, thorough information of upper extremity attachments was acquired [23,24]. The hamstring origins were also studied in detail this way [25]. However, as such a set-up is expensive and highly sensitive to calibration, barium sulfate can be injected to visualize ligaments in computed tomography (CT) images [26,27]. As a radiopaque paint, it offers an inexpensive alternative to mark attachments and reconstruct them during segmentation [28]. This allows for high accuracy by avoiding the distance between points of measurement. Moreover, the gold standard in musculoskeletal modeling is to offer corresponding points of functional muscle subunits [7,29] or to use generalized models [30]. This approach ignores the anatomical variation in location or morphology of individual attachment areas [21] and leads to inaccurate predictions from imprecise modeling [8] as it can affect the muscle moment arms [31,32], muscle force calculation, as well as joint dynamics [8,9,33]. Davico et al. [9] investigated the personalization of musculoskeletal models and showed that the musculoskeletal anatomy and muscle activation patterns, particularly, had a considerable influence on joint force calculation. 

Muscle attachments related to the patellofemoral and tibiofemoral sites are rarely studied extensively. Instead, investigators often focused on single muscles, evaluated muscle attachments in a simplified manner or did not focus on the individual differences in attachment areas [7,14,25]. In the present feasibility study, we introduced a reliable method to study several muscle attachments in order to reconstruct the attachment sites in 3D based on CT imaging for future applications in musculoskeletal modeling. We aimed to quantify the individual differences in muscle attachment areas for knee-related muscles.

## 2. Materials and Methods

### 2.1. Specimen Preparation

**Figure 1:**
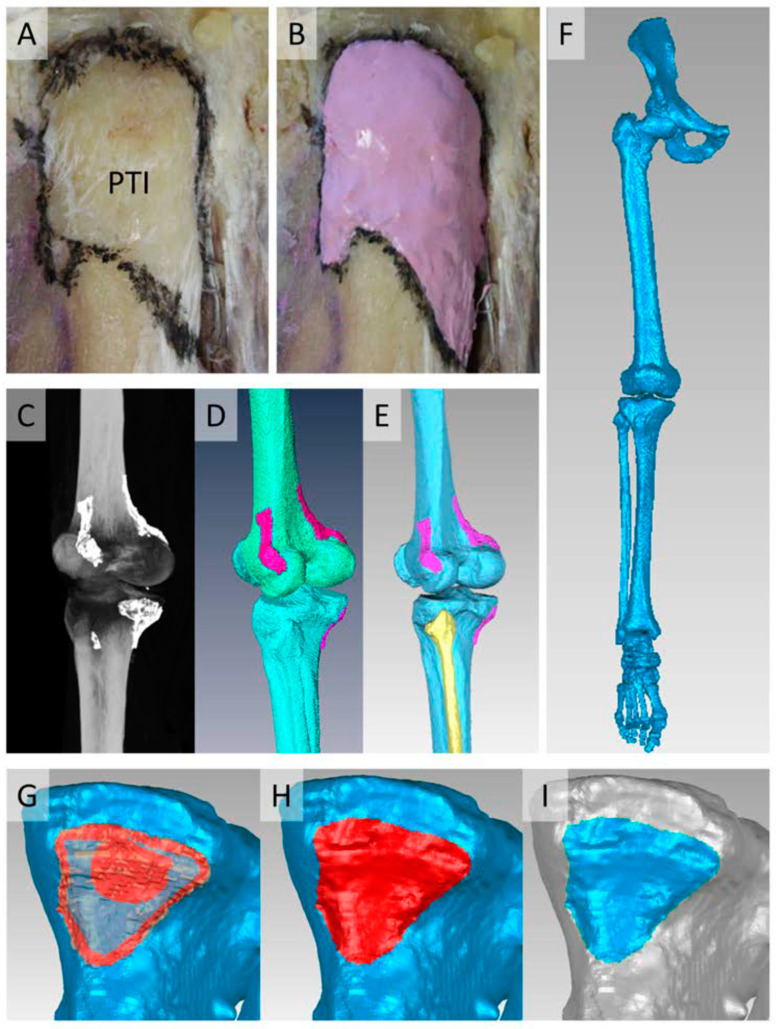
The process of reconstruction of bones and attachment markings is as follows: (**A**) Contour of the patellar tendon insertion (PTI) footprint at the anterior tibia after dissection by a surgical marker. (**B**) Painted footprint of the PTI with radiopaque paint. (**C**) Radiopaque markings of attachments during CT. (**D**) Rough surfaces after the segmentation process. (**E**) Smoothed and corrected bone surfaces with unedited paint surfaces. (**F**) Coherent model with all bones in position after fixation. (**G**–**I**) Process of footprint reconstruction. (**G**) Tracing of contact area through a transparent paint surface on the bone. (**H**) Fully traced contact area on a bone. (**I**) Generated footprint surface on the bone.

Four nonpaired, formalin-fixed human legs were used to prepare knee-related muscle attachment areas in four steps. This was approved by the local ethical committee of the University of Rostock (registration number A 2016-00083). Specimen preparation was performed by the authors assisted by medical prosectors. Attachment areas were freed of surrounding tissues by scissors and pulled off the bones. The outlines of the bony attachments were marked with a surgical marker and painted with a barium sulfate containing paint (60 g BaSO_4_ in 30 mL water and 10 mL acrylic paint; Figure 1A,B). 

The muscles origins (O) and insertions (I) were dissected step-wise (plantaris and popliteus muscles were not evaluated) and a CT scan (Brilliance CT Big Bore, Philips, Amsterdam, The Netherlands) was performed after each dissection with a slice thickness of 1 mm (varying between 716 to 1063 slices per scan) covering the following structures: CT1: M. semimembranosus (O&I), M. sartorius (O&I), M. gastrocnemius (O), M. rectus femoris (O); CT2: M. biceps femoris (O&I), M. gracilis (O&I), M. vastus medialis (O); CT3: M. semitendinosus (O&I), M. vastus lateralis (O); CT4: Patellar tendon (O&I), M. vastus intermedius (O), M. quadriceps tendon (I) and Achilles tendon (I).

In the case of multipart insertions (e.g., M. semimembranosus), attachments into ligaments or joint capsules were excluded. Possibly conjoined tendons were carefully separated along the fibers until the surface of the bone was reached (e.g., hamstring origins); the quadriceps tendon, however, was taken as one. The patella is tediously surrounded, nevertheless, quadriceps insertion and patellar tendon origin were separated based on the direction of the fibers when attached to the bony surface. A small area of less than 5 mm in width along the patella was left unpainted in between for distinction during segmentation.

### 2.2. Bone Reconstruction

The bone geometry and the adjacent highly radiopaque paint were reconstructed from CT datasets in AMIRA^®^ v.5.4.1 (v5.4.1, Zuse Institute Berlin, Berlin, Germany; Thermo Fisher Scientific, Waltham, MA, USA). The structures were segmented layer-by-layer using the image segmentation editor with an intensity threshold based on Hounsfield units (HU) (Figure 1C–E). As established, we used a primary HU value for bone of 250 up to 3000 [34]. Strongly calcified tendons were manually excluded (except in case of the quadriceps insertion). The surfaces of identical femur, pelvis, tibia and fibula were generated repeatedly, whenever an attachment was marked on it, to decrease inaccuracies of the labeling at the paint/bone contact area. The patella was labelled twice, with and without a mark. The foot was labelled only with a mark after CT4 since the maximal scanning length craniocaudally did not allow for a simultaneous scan from pelvis to foot. When segmentation was completed, 3D surfaces of the bones and the paint were reconstructed automatically using triangulated surfaces.

### 2.3. Generation of Coherent Models

These surface models were imported into GEOMAGIC studio v.13 (v2013, 3D Systems, Rock Hill, SC, USA) via a STL interface in ASCII mode. All bone surfaces were transformed based on the donor’s anatomy during CT1 and a 3D comparison was performed to evaluate the segmentation process. The different surfaces of the identical bones were merged, corrected (removal of holes and sharp edges) and smoothed, while the surfaces of the unmarked patella and the foot were only corrected and smoothed. Hence, one coherent 3D surface model for each leg was constructed (Figure 1F). Volume’s center of gravity, volume and surface area (SA) for every bone were calculated. Additionally, femoral head diameter (FHD), shaft diameter (SD), length of the mechanical axis (MA) and length of the transepicondylar axis (TEA) of the femur were measured. All data are expressed in the defined coordinate frame of the femur of each leg, with the center of the TEA as the origin, using a standardized coordinate system according to the International Society of Biomechanics definition [35]. Briefly, the y-axis is defined by the TEA midpoint and femoral head center point towards cranially, the z-axis lies perpendicular to the y-axis in the plane with the origin and femoral head center point, and the x-axis lies perpendicular to both other axes pointing anteriorly. 

### 2.4. Projection of Attachments on Bone Surfaces

The surfaces of the paint marks were aligned using the transformation matrices of the bones calculated previously for the construction of coherent models. In case of overlap between the attachment paint marks (e.g., hamstring origins, origins around the linea aspera, pes anserinus superficialis insertions), the later applied mark was slightly corrected. The contact area of the paint with the surface of the bones was manually traced on the bone in GEOMAGIC studio and generated as a separate surface (Figure 1G–I).

### 2.5. Muscle Data Collection

The footprint and centroid of each attachment area were calculated in GEOMAGIC studio. For use in future musculoskeletal modeling, a breakthrough point of the centroid was generated as the intersection point with the attachment surface of a line perpendicular to a best-fit plane of the attachment area through the centroid. In this manner, we created attachment points on the surface of the respective bones. Some muscle attachments needed further division for meaningful point generation. Linear attachments were divided three times in length from the most proximal to the most distal point. The vastus intermedius was separated six times: in half by a plane passing through the most proximal point, the most distal point, and the center point between the lateral and medial edges, and three times equally in length.Vastus medialis and vastus lateralis were separated in a superior part (polygonal shaped) and an inferior part (linear shaped, separated three times) by the anterior and distal margin of the trochanter minor, respectively. The fibular insertion of biceps femoris was separated by a plane through the coordinate origin, the centroid and the apex of the distal crest of the fibular head. If a single breakthrough point was still not determinable for the attachment unit, it was separated along the thinnest part. The tibial insertion of the biceps femoris of Specimen 3 was naturally separated into two areas, which were combined for evaluation.

### 2.6. Data Analysis

Results are expressed as mean with the standard deviation (STD) of the population and the corresponding coefficient of variation (CV). The deviations from the reference model of CT1 were calculated using GEOMAGIC studio. Furthermore, volume’s center of gravity, volume and surface area (SA) for every bone were calculated.

## 3. Results

### 3.1. Specimen Characterization

The four legs (three left, one right) were from nonadipose male donors with an age between 68 and 78 years and a height of 170 to 180 cm. Causes of death were acute myocardial infarct, heart failure and multiorgan failure after metastatic cancer. Previous diseases were of the spectrum of cardiovascular diseases, chronic obstructive pulmonary disease and diabetes mellitus. Musculoskeletal diseases were not known or detected; however, some tendon calcifications were observed (Supplementary Figure S1). Muscle or bone quality were not evaluated.

### 3.2. Segmentation Deviation Analysis

**Figure 2:**
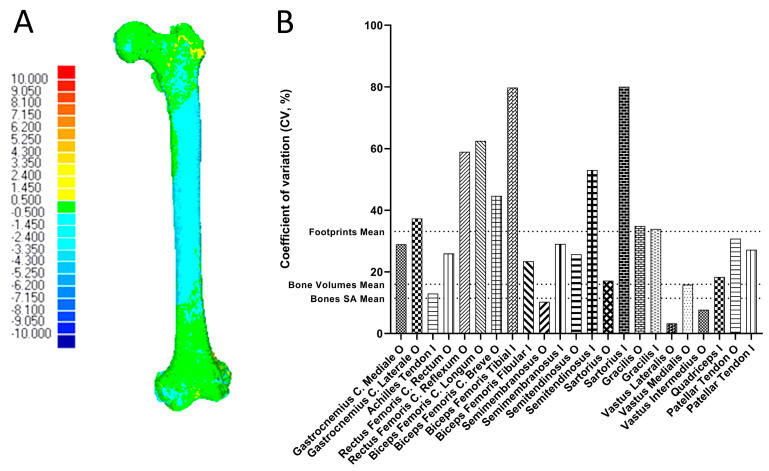
Exemplary deviation analysis of a femur and variation of muscle attachment footprints. (**A**) Deviation of the segmented surface of CT4 to the reference surface of CT1. Underneath the paint for the vastus intermedius footprint the surfaces deviate strongest because paint and bone surfaces interblend on the CT images. Scale expressed in mm. (**B**) Coefficient of variation (CV) of each muscle in relation to the mean CV of the bones’ volumes, surface areas and the mean CV of all muscles.

We calculated the deviations (mm) of each subsequent CT reconstruction from the reference surface of CT1 (Table 1). All segmented femoral surfaces had a mean deviation from the reference surface of 0.48 ± 0.14 mm, the pelvic surfaces of 0.40 ± 0.10 mm, the tibiofibular surfaces of 0.54 ± 0.15 mm and the patellar surfaces of 0.70 ± 0.15 mm; altogether, there was a deviation from the reference surfaces of 0.50 ± 0.16 mm. The highest deviation after segmentation was seen underneath the painted muscle attachment areas (Figure 2A).

### 3.3. Morphological Data

Volumes, SA and volume’s centers of gravity of the bones were calculated (Table 2). Volumes and SA had a mean CV of 15.94 ± 1.69% and 11.42 ± 1.55%, respectively. We measured femoral morphology (Table 3), evaluated muscle attachment areas and centroids (Table 4) and generated breakthrough points for each attachment site (Supplementary Table S1). Femoral morphological parameters varied around 7.38 ± 2.01% between individuals. On the contrary, the mean surface areas of the attachments showed large variations (Figure 2B); the mean CV for all attachment site areas was 33.12 ± 20.82%. Attachment areas with high variation (CV > 50%) were rectus femoris caput reflexum O (58.94%), semitendinosus I (53.03%) and sartorius I (80.05%). The insertions forming the pes anserinus superficialis (semitendinosus, sartorius, gracilis) varied from moderately to strongly (53.03%, 80.05%, 33.90%, respectively), which might indicate some kind of dependence from one another. The attachment areas with relatively low variation (CV < 15%) are the origins of semimembranosus (10.32%), vastus lateralis (3.29%) and vastus intermedius (7.74%) as well as the Achilles tendon I (12.94%).

## 4. Discussion

The focus of our present study was to establish an accessible and inexpensive method to evaluate muscle attachment sites to construct a database of individual attachment morphologies. Furthermore, the aim was to quantify individual differences of attachment areas of knee-related muscles. In our feasibility study, we showed a high diversity of muscle attachment sites in size and location, mostly exceeding the individual variations in bone morphology. We analyzed the segmentation deviation and found a low deviation of 0.50 ± 0.16 mm. The bones around the dense cortex sometimes shared HU with the paint. Labeling within the contact area in these cases is difficult and prone to minor errors but does not alter the size and location of attachments. In a previous study [34], the 3D segmentation process was verified in an inter-laboratory study. Using different software packages and algorithms, the participating groups independently extracted the 3D geometry of a single human femur from CT data. Four different segmentation software packages were used: AMIRA^®^ (FEI Visualization Sciences Group, Hillsboro, OR, USA), Mimics^®^ (Materialise N.V., Leuven, Belgium), YaDiv (Welfenlab, Leibniz Universität Hannover, Hannover, Germany), and Fiji Life-Line. Stereolithography files were imported into GEOMAGIC studio v.2013 (Raindrop Geomagic, Triangle Park, NC, USA). They found no crucial differences compared with an optical scan of the original surface [34].

We investigated the muscle attachment sites of formalin-fixed human leg specimens. Herein, we analyzed the locations and areal shapes of the lower extremity muscles spanning the knee joint. We identified the variance of locations and shapes of the attachments of the different specimens. Therefore, the present study provides important insights into individual differences in musculoskeletal geometry based on methodology to identify the muscle attachment areas using a CT scan. A geometry dataset is provided for musculoskeletal modeling, similar to [7], but with different specimens and varying muscle attachment areas. Based on medical imaging data, we considered a more inter-individual anatomical variability in size and shape of the attachment footprints. The implementation of these attachment sites into a musculoskeletal multibody model requires the definition of an origin and insertion point. For this purpose, we calculated breakthrough points.

In addition, the influence of the varying muscle attachment areas on the predicted muscle forces can be investigated in future studies by means of inverse dynamics as these parameters have a considerable effect in model predictions [8,36]. To our knowledge, there are rarely sensitivity analyses that address this issue since there is no reliable numeric information for the areal shape and size of each of the individual muscle subunits. Fukuda et al. [21] investigated the differences in muscle attachment site for the hip muscles using an optical tracker. Contrarily, our feasibility study investigated the variation in the muscle attachment area of knee-related muscles in different specimens, providing the possibility to perform sensitivity studies. Future studies could investigate the impact on musculoskeletal model prediction, e.g., quadriceps force or tibiofemoral contact force, by perturbation of the origin and insertion points within the respective muscle attachment area [36].

Nevertheless, there are some limitations in the present study. We did not include muscle volumes and subcutaneous fat. Furthermore, we used a limited sample size, which did not cover all anatomical variations of human legs. In this context, female geometries should especially be integrated into future studies as presented by Andreassen et al. [20]. Moreover, muscle architecture can also be changed besides the musculoskeletal geometry [37]. The technique of marking the muscle attachment sites with barium paint and the use of a CT scanner are time-consuming as each muscle was outlined manually with a surgical marker and each leg was scanned and segmented four times. Nevertheless, given the inexpensive nature of the radiopaque paint, this method is a very cost efficient and easy-to-implement technique.

### Variations of Muscle Attachments

Literature about attachment dimensions is sparsely found. Some studies described specific anatomical components but a coherent analysis of the diverse muscular aspects affecting knee function is missing. The variation of footprints is of great relevance for the musculoskeletal geometry, especially muscle moment arm as one of the most sensitive parameters [7]. Further studies are needed to investigate the practical consequences of our presented results in musculoskeletal modeling. Particularly, the errors we might expect when we incorporate our data into a musculoskeletal model of the lower extremity. Depending on the attachment points, muscle moment arms and muscle–tendon lines of action differ, thus changing the predicted muscle forces. Consequently, differences in moment arms lead to inaccuracies in the prediction of muscle and joint contact forces [8,9,33]. Such joint contact forces contribute to the progression of joint arthritis [38]. It was also shown that considering subject-specific bone structures and attachment areas leads to more accurate joint dynamics predictions [9]. 

Despite variations in methodology, studies investigating specific muscle attachments are largely consistent with our results [28,39,40]. The hamstring origin complex is a particularly well-studied attachment site due to the high prevalence of hamstring injuries in sports [28]. The origins of biceps femoris caput longum and semitendinosus usually attach as a strong conjoined tendon, therefore being particularly sensitive to preparation methods. Compared to the study by Philippon et al. (2015), our mean overall hamstring origin was larger with 1336.52 mm^2^ vs. 991.70 mm^2^, due to a greater conjoined tendon footprint of 864.74 mm^2^ vs. 567.00 mm^2^, while the origins of the semimembranosus were alike (471.78 mm^2^ vs. 412.40 mm^2^ by Philippon) [25]. 

The semimembranosus insertional attachment is less investigated despite the relevance of the tendon for the posteromedial corner of the knee and related injuries. The insertion tendon is a complex structure with slips inserted into the medial collateral ligament, the oblique popliteal ligament and a groove on the posteromedial aspect of the tibia named pes anserinus profundus. The arrangement of these insertion slips was previously analyzed [12,14]. Fukuda et al. [21] quantified the differences in the muscle attachment areas for hip-related muscles for eight human specimens using an optical tracker but did not provide insertion points using this dataset. To our knowledge, no study quantified the insertional footprint of the knee-related muscle considering its polygonal shape. A similar complex structure is the pes anserinus superficialis including the insertions of semitendinosus, sartorius and gracilis. The distal tendons of these muscles are regularly used as autografts for ligament reconstructions [41], but morphological quantification data of the attachment area is largely missing. In our study, the sartorius insertion showed high variation, something allured to be due to a highly varying inferior part [13,42]. 

The quadriceps group, the main antagonist of the hamstring group and extensor of the knee, and the gastrocnemius with the Achilles tendon are related to knee dysfunctions and common sports injuries [4,43], but attachment morphologies are unstudied. Ryan et al. (2014) measured the widths and lengths of the rectus femoris heads attachments [44]. Given the substantial variations seen in these sites, comparing the study (*n* = 6) with ours (*n* = 4) is difficult. The Achilles tendon was studied in descriptive ways [11,40] and reported with a similar mean area and variation to ours (18% vs. 13%) [40].

In summary, our results are largely consistent with the few previously conducted studies. Since the number of studies is small and the methods differ, more data is needed to assess physiological footprint dimensions sufficiently. However, we present the first step in establishing a thorough database of individual attachment morphologies. Our feasibility study had some limitations: the method was recently established and may be more prone to error than 3D-digitizer and the number of specimens evaluated in this study was small. For a more thorough analysis, however, a larger cohort is needed.

## 5. Conclusions

Depending on morphology and location, we found large variations regarding attachment dimensions within our limited number of human specimens. We assume that lifestyle or genetics notably influence the shape and size of muscle attachment sites. Future studies should, therefore, include environmental factors and patient’s lifestyles and gender in their investigations of muscle attachments and we would recommend increasing the impact of individuality in biomedical engineering research. Nevertheless, to have representative results for the young population that is more concerned with related pathologies, studies with similar elaborateness but nonterminal methodology need to be conducted. New, more advanced imaging methods like spectral CT that are able to characterize musculoskeletal pathologies noninvasively may be able to evaluate muscle attachment areas in this population in the future [45]. Since we processed the 3D data for musculoskeletal modeling, we want to progress the application of the muscle attachment data in respective musculoskeletal simulations. This study provides a technique for an anatomical description of the muscle attachment sites for knee-related muscles that can be used in future numerical studies. This may help to determine variability of the anatomical positions of muscle attachment sites, which is useful for orthopedic surgeons and computational biomechanics.

## Figures and Tables

**Table 1:** Deviations of the segmented surfaces from the references surface after CT1 expressed in mm.

Specimen	Femur CT2	Femur CT3	Femur CT4	Pelvis CT2	Pelvis CT3	Tibia CT2	Tibia CT3	Tibia CT4	Patella CT4
1	0.38	0.38	0.61	0.52	0.60	0.63	0.34	0.68	0.60
2	0.40	0.45	0.71	0.40	0.39	0.47	0.45	0.49	0.92
3	0.37	0.34	0.66	0.31	0.32	0.69	0.71	0.74	0.74
4	0.34	0.39	0.64	0.32	0.34	0.35	0.30	0.65	0.54
Mean	0.37	0.39	0.66	0.39	0.41	0.53	0.45	0.64	0.70
STD	0.02	0.04	0.04	0.08	0.11	0.13	0.16	0.09	0.15

**Table 2:** Morphological bone parameters.

Bones	Volumes [mm^3^]		Surface Area [mm^2^]		Volume’s Center of Gravity [mm]		
	Mean ± STD	CV [%]	Mean ± STD	CV [%]	Mean x ± STD	Mean y ± STD	Mean z ± STD
Pelvis	3.93 × 10^5^ ± 5.32 × 10^4^	13.54	7.11 × 10^4^ ± 6.21 × 10^3^	8.74	16.92 ± 6.27	454.79 ± 40.49	7.12 ± 4.32
Femur	6.45 × 10^5^ ± 1.11 × 10^5^	17.23	7.20 × 10^4^ ± 8.77 × 10^3^	12.17	5.75 ± 3.10	198.85 ± 24.32	16.72 ± 2.24
Patella	2.41 × 10^4^ ± 4.18 × 10^3^	17.38	4.76 × 10^3^ ± 5.64 × 10^2^	11.85	49.73 ± 2.12	6.49 ± 3.72	4.23 ± 0.52
Tibia/Fibula	3.58 × 10^5^ ± 6.21 × 10^4^	17.33	6.71 × 10^4^ ± 8.99 × 10^3^	13.40	13.31 ± 9.16	167.38 ± 12.12	8.77 ± 5.64
Foot	2.45 × 10^5^ ± 3.49 × 10^4^	14.24	6.03 × 10^4^ ± 6.58 × 10^3^	10.92	44.91 ± 34.53	454.51 ± 42.08	28.18 ± 21.02

**Table 3:** Established morphological dimensions and axis of the femur.

Specimen	Femoral Morphological Parameter [mm]			
	MA	TEA	FHD	SD
1	430.40	97.80	50.60	36.60
2	403.70	95.00	54.70	40.40
3	442.10	88.50	51.30	34.90
4	345.30	85.30	47.10	30.90
Mean	405.38	91.65	50.93	35.70
STD	37.37	4.98	2.70	3.41
CV [%]	9.22	5.44	5.30	9.56

**Table 4:** Surface area, coefficient of variation and mean centroid of the muscle attachment footprints.

Muscle Attachment	Surface Area [mm^2^]	CV [%]	Centroid [mm]		
	Mean ± STD		Mean x ± STD	Mean y ± STD	Mean z ± STD
Gastrocnemius Caput Mediale O	630.12 ± 182.71	29.00	5.41 ± 1.72	24.21 ± 3.96	19.97 ± 3.51
Gastrocnemius Caput Laterale O	788.24 ± 293.77	37.27	2.89 ± 2.14	19.10 ± 8.41	35.02 ± 3.09
Achilles Tendon I	1000.21 ± 129.47	12.94	114.80 ± 41.42	417.36 ± 41.47	27.23 ± 17.40
Rectus Femoris Caput Rectum O	282.42 ± 73.21	25.92	40.90 ± 4.76	441.16 ± 39.09	4.05 ± 2.94
Rectus Femoris Caput Reflexum O	366.77 ± 216.17	58.94	4.90 ± 2.71	441.88 ± 40.23	18.03 ± 1.32
Biceps Femoris Caput Longum O	260.42 ± 162.77	62.50	62.22 ± 8.87	381.46 ± 41.80	7.38 ± 7.11
Biceps Femoris Caput Breve O	582.20 ± 259.99	44.66	2.71 ± 2.26	144.29 ± 17.82	19.04 ± 0.56
Biceps Femoris Tibial I	103.86 ± 82.87	79.79	8.13 ± 5.24	46.70 ± 4.90	43.34 ± 3.49
Biceps Femoris Fibular I	401.88 ± 94.16	23.43	22.57 ± 6.19	52.71 ± 6.37	48.95 ± 4.33
Semimembranosus O	471.78 ± 48.69	10.32	49.83 ± 9.45	370.54 ± 41.35	5.23 ± 4.93
Semimembranosus I	1048.10 ± 304.77	29.08	16.88 ± 3.21	55.22 ± 1.86	21.42 ± 0.73
Semitendinosus O	604.32 ± 155.25	25.69	64.19 ± 10.99	360.41 ± 41.71	10.73 ± 10.21
Semitendinosus I	226.50 ± 120.11	53.03	11.50 ± 5.61	97.74 ± 10.52	7.08 ± 4.02
Sartorius O	115.94 ± 19.92	17.18	62.48 ± 5.84	472.53 ± 39.85	9.45 ± 8.04
Sartorius I	313.18 ± 250.69	80.05	14.28 ± 3.34	86.16 ± 18.82	5.06 ± 4.82
Gracilis O	132.23 ± 46.13	34.88	16.69 ± 12.57	360.09 ± 34.53	79.06 ± 4.38
Gracilis I	67.18 ± 22.78	33.90	14.80 ± 6.41	85.25 ± 5.92	4.76 ± 3.73
Vastus Lateralis O	2493.56 ± 82.11	3.29	5.53 ± 3.11	238.36 ± 54.33	19.55 ± 8.51
Vastus Medialis O	3410.55 ± 541.07	15.86	1.54 ± 1.36	301.81 ± 35.94	50.51 ± 4.47
Vastus Intermedius O	10,968.48 ± 849.01	7.74	24.07 ± 4.15	224.31 ± 22.81	31.96 ± 2.28
Quadriceps Tendon I	1480.95 ± 271.94	18.36	56.66 ± 2.42	10.52 ± 6.66	4.08 ± 0.65
Patellar Tendon O	992.16 ± 305.28	30.77	50.81 ± 1.95	15.20 ± 6.47	5.20 ± 1.82
Patellar Tendon I	733.14 ± 198.92	27.13	24.11 ± 5.86	73.44 ± 8.94	16.76 ± 6.60

## Data Availability

The datasets used during the current study are available from the first author on reasonable request.

## Supplementary Materials

The following supporting information can be downloaded at: https://www.mdpi.com/article/10.3390/life14060778/s1, Figure S1: Pathologies of specimen. [A] Calcification of the hamstring tendon (and sacrotuberous ligament, excluded from segmentation) and [A’] on the patella. [B] Patella dysplasia with medial trochlear hypoplasia. [C] Broken acetabulum rim, possibly postmortem; Table S1: Generated breakthrough points for muscle attachments and subunits (O = origin, I = insertion).
