# Supplementary material for: Evaluation of 3D Footprint Morphology of Knee-Related Muscle Attachments Based on CT Data Reconstruction: A Feasibility Study
Source: Life (Basel). 2024 Jun 19;14(6):778. doi: 10.3390/life14060778 (PMC11204608; doi:10.3390/life14060778)
Supplement: Supplementary file 1 [file life-14-00778-s001.zip › Neumann_et_al_Supplementary_Captions.docx]

Supplementary Captions

Figure S1: Pathologies of specimen. [A] Calcification of the hamstring tendon (and sacrotuberous ligament, excluded from segmentation) and [A'] on the patella. [B] Patella dysplasia with medial trochlear hypoplasia. [C] Broken acetabulum rim, possibly post-mortem.

Table S1: Generated breakthrough points for muscle attachments and subunits (O=origin, I=insertion).
