# Supplementary figures and images for: Evaluation of 3D Footprint Morphology of Knee-Related Muscle Attachments Based on CT Data Reconstruction: A Feasibility Study
Source: Life (Basel). 2024 Jun 19;14(6):778. doi: 10.3390/life14060778 (PMC11204608; doi:10.3390/life14060778)

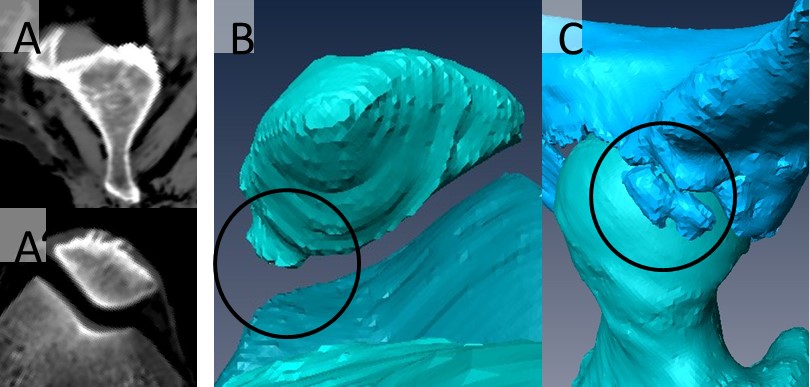

Supplement: Supplementary file 1 [file life-14-00778-s001.zip › Supplementary Figure S1.jpg]
